# Ingestion of Partial Denture after General Anesthesia Induction and Ventılation: A Rare Case Report

**DOI:** 10.1155/2019/8726508

**Published:** 2019-05-29

**Authors:** Hakan Akelma, Fikret Salik, Ömer Başol, Hüseyin Bilge, Akif Yıldırım, Şule Özgün

**Affiliations:** ^1^Health Sciences University, Gazi Yaşargil Training and Research Hospital, Anesthesiology and Reanimation Clinic, Diyarbakir, Turkey; ^2^Health Sciences University, Gazi Yaşargil Training and Research Hospital, General Surgery and Burn Clinic, Diyarbakir, Turkey

## Abstract

The swallowing of dentures during general anaesthesia is a significant problem for anesthesiologists. It is seen more often in patients with psychiatric disorders, mental retardation, alcoholism, or poor quality dentures. It has become an important issue for anesthesiologists preoperatively due to the increase in the proportion of dentures associated with prolongation of life. In elderly, the use of partial fixed prosthesis increases and the risk of swallowing dentures is increased. In this case report, it was presented that the denture was swallowed spontaneously before intubation while the patient was ventilated preoperatively.

## 1. Introductıon

The swallowing of prostheses is an important problem for general surgery, gastroenterology, anesthesiology, psychiatry, neurology, and emergency medicine. In adults, swallowing foreign bodies is seen less frequently. However, it is seen more often in adults with psychiatric disorders, mental retardation, alcoholism, or poor quality dentures [1]. Approximately 80% of foreign bodies in the digestive system pass spontaneously from the digestive tract without any complications in the whole gastrointestinal system. In 10%-20% of patients, endoscopic intervention is required without surgery, and in 1% or less, an operation is necessary [2, 3]. The most common localization of swallowed dentures is the oesophagus [4], and it is rare for them to be reported in the small intestine. The most commonly seen surgical complications are perforation, bleeding, and obstruction.

Most anesthetists work from the mouth. To prevent perioperative dental damage and complications, it is important to determine the dental status of the patient and identify any sensitive teeth or soft tissues and the anesthesia risk factors associated with these in a full preoperative evaluation. Nowadays, many patients have dentures which are more fragile than the natural teeth. The increased use of partially fixed dentures in the elderly increases the possibility of swallowing prostheses. This possibility is often overlooked. If it is not noticed that the patient has partial dentures, there is a possibility that the denture will be pushed into the oesophagus and swallowed during ventilation with positive pressure following preoperative induction. This is a rarely seen complication for anaesthetists.

In this paper, the uncommon case is presented of a patient who swallowed a denture which dislodged as a result of the positive pressure created during preoperative ventilation and in the postoperative period, it spontaneously passed through the intestinal canal without any harm to the patient.

## 2. Case

**Figure 1:**
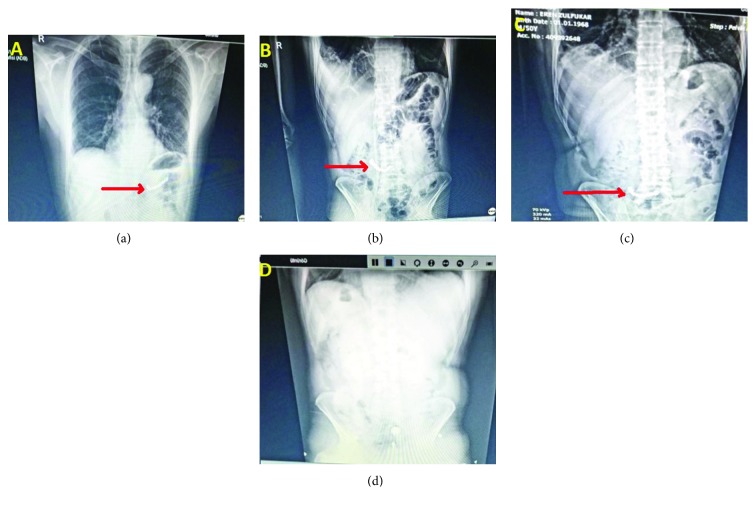
Daily radiological follow-up of swallowed dentures. (a) Day 1: dentures seen in the stomach antrum. (b) Day 2: dentures seen in the jejunum. (c) Day 3: dentures seen in the ileocecal region. (d) Day 5: dentures not observed on the abdominal radiograph.

A 50-year-old male presented at the General Surgery Polyclinic due to increased complaints of swelling and intermittent pain in the neck which had been ongoing for 6 years. On the neck ultrasound imaging, heterogeneous nodules were observed in the parenchyma of 27 × 19 mm in the right lobe and 20 × 16 mm in the left lobe and central anechoic cystic nodules 40 × 18 mm at the isthmus level extending towards the lumen. Thyroid function tests were normal and the patient was diagnosed with multinodular goitre. The patient had no comorbid disease and after premedication was admitted to the operating room for elective surgery. Monitorization was applied on the operating table: TA: 130/85 mmHg, pulse: 75 bpm, SpO_2_: 99%. Sedation was administered by 2 mg midazolam, then anaesthesia induction was made with 2-3 mg/kg propofol, 100 mcg fentanyl, and 0.6 mg/kg rocuronium. After mask ventilation for 3-5 mins, the patient was intubated with no problems. No hemodynamic or respiratory problems were experienced in the intraoperative period. The surgery lasted 1 hr and 45 mins, after which the patient was awakened with no problems and was transferred to the postoperative anaesthesia care unit (PACU). When the patient was fully awake, he was experiencing chest pain and complained that dentures were not in place in his mouth, so a posterior-anterior pulmonary radiograph was taken and a standing direct abdominal radiograph. The dentures were observed in the stomach (Figure 1(a)). In the history taken from the patient, it was seen that when going to the operating room, partial fixed dentures were in place, and he stated they were in his mouth until reaching the operating room. The emergency gastroenterologist was consulted, and the patient was evaluated but as he had already eaten food, endoscopy procedures were postponed until the following day. In the upper gastrointestinal endoscopy applied the following day, despite having passed the ligament of Treitz, the dentures could not be visualized. On the standing direct abdominal radiograph, the dentures were seen to be in the jejunum (Figure 1(b)). The abdominal examination was comfortable for the patient, so it was decided to continue medical follow-up. A standing direct abdominal radiograph was taken daily (Figure 1(c)), and on the radiograph taken on the 5th day, the dentures could not be seen (Figure 1(d)). The patient was questioned. The dentures had been expelled during bowel evacuation, but the patient did not recall anything other than a slight pain during evacuation.

## 3. Discussion

Foreign bodies in the aerodigestive tract are not an uncommon problem, especially in the paediatric population. However, they are not frequently seen in adults. Foreign bodies that are most commonly seen in the digestive system in adults are chicken and fish bones in Asia and large pieces of food in western countries [4].

The most common foreign body in adults requiring surgery is a needle, and those causing gastrointestinal perforation (generally, the small intestine) and requiring surgery are fish bones (68% of all foreign bodies causing small intestine perforation).

Dentures which are used as medical prosthetic devices for chewing and for aesthetic reasons or to develop self-confidence are a particular type of foreign body. Aspiration and swallowing of this foreign body is generally seen in patients with psychoneurological deficit, alcohol poisoning, drug overdose, under general anaesthesia, or with maxillofacial trauma. Another significant factor increasing the risk of swallowing dentures is a lack of awareness of the patients of changes in the dentures and incompatibility with the jaw at regular check-up examinations [5]. In addition to early diagnosis and treatment of the swallowing of dentures, it is key that there is awareness of situations that can stimulate prevention. Dental practitioners are at the forefront of prevention of this potential problem, but ear, nose, and throat specialists, surgeons, and anaesthesia specialists must also be aware of this situation.

Acute impairments of consciousness (craniofacial trauma, stroke, convulsions, and poisoning) increase the risk of tooth aspiration or swallowing and aspiration of the stomach contents. In conscious patients, activities in which dentures could be swallowed or aspiration could occur have been well-documented [5], including falls, eating, drinking, sleep, and anaesthesia [6]. To prevent accidental loss or damage, any removable prosthesis (e.g., prostheses, orthodontic devices) or piercings of the lips or tongue soft tissue must be removed before anaesthesia induction, labelled, and stored safely.

The main contraindication for endoscopic removal of a swallowed prosthesis is evidence of high risk or primary or secondary complications. Foreign bodies affecting the hypopharynx can be removed with hypopharyngoscopy and direct laryngoscopy [7]. However, the development of open surgery complications such as transcervical oesophagotomy, transthoracic oesophagotomy, or oesophagectomy is a significant cause of failure for these procedures [5].

Prostheses affecting the small and large intestines can be removed endoscopically or with surgery (laparotomy or laparoscopy) depending on the size, configuration, risk of intestinal wall perforation, or any complication [8]. In the current patient, the swallowing of the dentures was seen during anaesthesia sedation, which is an uncommon event. A previous case has been reported where a partial fixed denture was swallowed and was subsequently recovered with endoscopy during general anesthesia. In the current patient, the loss of the denture was noticed in PACU and despite preparations made for endoscopy, this procedure could not be applied immediately as the patient had eaten food.

The majority of cases reported in literature have been seen to be the ingestion of partially removable prostheses in particular. However, it has also been reported that when fixed prostheses have been dislodged spontaneously or traumatically, they have been swallowed.

Complications of prosthesis ingestion include necrosis, perforation, penetration of adjacent organs, bleeding, and obstruction. Ulceration and vascular erosion result from digestive system bleeding, and reported cases have shown oesophageal and gastric bleeding caused by a swallowed prosthesis. As the large intestine has a wider diameter and its contents are more solid in texture, these help to prevent trauma to the intestinal wall, and so the entrapment and possible results of an ingested denture are rare. Theoretically, a denture which has passed the ileocecal valve should pass through the large intestine. If it becomes embedded, the only reason for this implantation of the denture can be colonic stenosis such as colon cancer. Several cases have been reported of sigmoid colon perforation [9]. In the current case, the swallowed denture was passed during defecation without any problem on the 3rd day and without the patient even noticing, which occurred as observed on the direct abdominal radiographs taken daily.

## 4. Conclusion

Dentures are a special type of foreign body requiring the awareness primarily not only of anaesthetists but also of other specialists. Fixed prostheses, especially those which are unstable, are at risk of aspiration and ingestion just as removable prostheses are. Early diagnosis and treatment is of vital importance in the management of swallowed prostheses. Loose, removable dentures, or unstable fixed prostheses must be well evaluated preoperatively. Removable prostheses must be removed before the transfer to the operating theatre. If an unstable, loose fixed prosthesis is noticed, it should be recommended that a dentist is consulted by the anesthetist evaluating the patient.
